# Can prophylactic HPV vaccination reduce the recurrence of cervical lesions after surgery? Review and prospect

**DOI:** 10.1186/s13027-023-00547-2

**Published:** 2023-10-29

**Authors:** Ling Han, Bingyi Zhang

**Affiliations:** 1 Department of Obstetrics and Gynecology, The First College of Clinical Medical Science, China Three Gorges University, Yichang City, Hubei Province People’s Republic of China; 2 Department of Obstetrics and Gynecology, Yichang Central People’s Hospital, Yichang City, Hubei Province People’s Republic of China; 3 Department of Ultrasound Imaging, The First College of Clinical Medical Science,, China Three Gorges University, Jiefang Road 2, Yichang City, 443003 Hubei Province People’s Republic of China; 4 Department of Ultrasound Imaging, Yichang Central People’s Hospital, Jiefang Road 2, Yichang City, 443003 Hubei Province People’s Republic of China

**Keywords:** HPV, Vaccination, Cervical lesions, HSIL, CIN

## Abstract

Women with HSIL typically undergo conization/LEEP to remove cervical lesions, but the risk of HSIL lesions returning after surgical treatment remains higher than in the general population. HPV vaccination is essential to prevent cervical cancer. However, the effect of prophylactic HPV vaccination on reducing the risk of recurrent cervical lesions after surgical treatment remains unclear. This review aims to analyze and summarize the latest literature on the role of prophylactic HPV vaccine in reducing the recurrence of cervical lesions after surgery in patients with HSIL, and to review and update the history, efficacy, effectiveness and safety of HPV vaccine, focusing on the current status of global HPV vaccine implementation and obstacles.

## Introduction

The majority (> 95%) of cervical cancers are associated with persistent infection with high-risk human papillomavirus (HPV) [1]. In 2020, the World Health Organization reported that there were more than 600,000 new cases and 340,000 deaths worldwide every year, and more than 85% of the cases and deaths occurred in low- and middle-income countries [2, 3]. There were 110,000 new cases of cervical cancer in China, accounting for 18% of the new cases in the world [4]. Despite advances in cervical cancer screening and prevention in recent years, cervical cancer remains one of the most common gynecological tumors worldwide [5, 6]. More than 100 countries worldwide now include HPV vaccination in their routine prevention programs [7–9]. HPV vaccination is the lowest-cost public health measure against cervical cancer and a key measure to prevent invasive cervical cancer [10]. There are currently three types of vaccines available (bivalent, quadvalent, and ninvalent), all of which target at least two of the most carcinogenic virus genotypes (HPV 16, 18) [11, 12]. HPV vaccine has the best effect before first sexual intercourse. To ensure the effectiveness of the vaccine, women aged 11–12 should receive routine preventive vaccination [13].

There are two grades of squamous intraepithelial lesions in the 5th edition of WHO Classification of Tumors of female reproductive organs: low-grade squamous intraepithelial lesion (LSIL), including ervical intraepithelial neoplasia(CIN) 1, high-grade squamous intraepithelial lesions (HSIL), including ervical intraepithelial neoplasia2, 3 (CIN2, CIN 3). The primary objective of the cervical screening program is the early detection and treatment of precancerous lesions, namely low-grade squamous intraepithelial lesions (LSIL) and high-grade squamous intraepithelial lesions (HSIL). Women with high-grade squamous intraepithelial lesions(HSIL) typically undergo cervical conization to remove cervical lesions and prevent progression [14]. Although eliminating high-risk human papillomavirus (HPV) is not a treatment goal, many such women have achieved elimination of infection [15, 16]. While the effectiveness of resection surgery has been clearly demonstrated, the overall risk of recurrence of cervical lesions after cervical intraepithelial neoplasia(CIN) 2 + surgery is approximately 10–14%, and the risk of recurrence of CIN 3 + and CIN 2 + after 5 years is 6% and 16.5% respectively [17]. The women had a higher risk than the general population of developing invasive cervical cancer years after treatment [18]. There is growing evidence that HPV vaccination in women who have been treated with HSIL may reduce the recurrence rate of cervical lesions. There is increasing evidence that vaccination with the HPV vaccine in women with HSIL who have undergone conization may reduce the recurrence rate of cervical lesions [19–24]. The rationale behind the efficacy of the HPV vaccine as an adjunct remains unclear. So far, several hypotheses have been proposed to explain how women who are already infected might benefit from HPV vaccination. It has been hypothesized that HPV vaccination stimulates local antibodies that increase the immune response, blocking the entry of the virus into uninfected cells in the basal layer and preventing disease recurrence [24]. It has also been hypothesized that surgical treatment may reduce local inflammatory responses, induce higher intensity and longer-lasting local cellular immunity, and restore HPV naive microenvironment. The HPV vaccine is theoretically effective in preventing persistent and recurrent HPV infection, but the potential role of prophylactic HPV vaccination as adjunctive therapy following surgical removal has not been demonstrated [25–30]. It is clinically important to explore whether post-treatment HPV vaccination is associated with a reduced risk of CIN. Therefore, The aim of this paper is to analyze and summarize the latest literature on how prophylactic HPV vaccination can help reduce the risk of recurrence after CIN surgery, and to review and update the history, efficacy, effectiveness, and safety of HPV vaccines, with a focus on the current status of global implementation of HPV vaccines and the obstacles they face.

## HPV and cervical carcinogenesis

HPV is a small, non-encapsulated, double-stranded DNA virus that can infect mucosal and skin epithelial cells. The DNA genome of HPV encodes approximately eight open reading frames (ORFs). ORF is divided into three functional areas, including early area (E), late area (L), and non-coding part or long control area (LCR). The E region gene encodes proteins E1–E7 necessary for viral replication and is involved in the pathogenicity of the virus. The L region genes encode the capsid proteins L1 and L2 required for virion assembly, and the long control region (LCR) regulates gene expression and replication [31]. Most genital HPV infections are acquired through sexual contact, through which HPV can cause minor cervical damage. Subsequently, HPV penetrates into basal layer cells mediated by heparin sulfate proteoglycans and integrins (α6, β1, and β4) in basal epithelial cells [32, 33]. When basal cells are infected with HPV, heparin sulfate proteoglycans and integrins divide, remaining in the basal layer and retaining their ability to divide, acting as reservoirs for viral replication. When the HPV genome is transferred to the nucleus, the expression of the early HPV genes E1 and E2 is activated. E1 is an ATPase helicase. E1 and E2 are involved in viral replication and transcriptional regulation. E2 maintains the dissociation of the HPV genome through interactions with other cytokines. E2 also regulates the transcription of the cancer proteins E6 and E7. The E1 and E2 complexes interact with the ori locus in the long control region, which is thought to be the starting point for HPV DNA replication [34, 35]. E2 protein recruits E1 protein (a viral DNA helicase) to the binding site of the origin of viral replication and activates viral DNA replication [36, 37]. To ensure that cervical cells continue to grow and divide, the early HPV genes E5, E6 and E7 are expressed. E6 and E7 are small proteins with approximately 150 and 100 amino acids, respectively. They disrupt cell cycle checkpoint control by degrading cell cycle regulators and inhibiting CDK inhibitors (p21, p27), thereby evading host immune responses. E6 and E7 can promote the immortalization of cancer cells by activating the hTERT promoter through a C-myc-dependent mechanism [38, 39]. The expression of E4, L1 and L2 is activated after differentiation of basal cells. The viral genome then encodes the synthesis of L1 and L2 capsid proteins, which assemble into mature virions that are secreted from epithelial cells and allow the infection to spread [40]. During viral DNA replication, each cell has at least 1000 copies of the virus, which increases the expression of L1 and L2 capsid proteins and the assembly of infectious viruses [41].

Most women infected with HPV rely on their own immunity to clear the virus within a few years, and approximately 10–20% of women continue to be infected [41]. The persistence of HPV infection is due to the following mechanisms: (1) Malignant transformation caused by down-regulation of cell cycle control mediated by HPV cancer proteins and genetic damage. (2) High-risk papillomavirus develops multiple immune escape mechanisms to promote its invasion and prevent its recognition by host immune cells. Persistent HPV infection can lead to cervical dysplasia, CIN and even invasive cervical cancer [40, 42].

## Prophylactic vaccines

The prophylactic HPV vaccine is effective in reducing the incidence of HPV infection and HPV-related diseases, and there are currently three licensed vaccines available [43]. The bivalent vaccine (Cervarix), approved in 2007, targets two types of HPV16 and HPV18, which are potentially carcinogenic. The quadrivalent vaccine (Gardasil), approved in 2006, targets HPV16 and 18, as well as low-risk types of HPV11 and 6 that cause genital warts. The nonavalent vaccines (Gardasil 9) against HPV6, 11, 16, 18, 31, 33, 45, 52, 58was approved in 2016. HPV vaccines are made from purified L1 proteins using recombinant DNA technology. L1 protein self-assembles to form HPV-specific shells (Virus-like particles; VLP), which is structurally similar to HPV but does not contain any active products or DNA, and is therefore considered non-carcinogenic and non-infectious, and less harmful to humans than vaccines made from attenuated HPV genomes [44]. After the prophylactic HPV vaccine is introduced into the human body, the antigen portion of the vaccine VLP binds to antigen presenting cells (APC) and then presents the antigen to T cells, which have a variety of functions and can differentiate into several T cell lineages, including cytotoxic T cells, T helper cells or memory T cells. T helper cells in turn stimulate naive B cells to become plasma or memory B cells [45]. Long-lived plasma cells (LLPCS) produce and secrete antigen-specific antibodies after vaccination, thus allowing circulating antibodies to persist and also aid in quick recall when encountering HPV antigens again [46]. As a result, LLPC, memory B cells, and T cells prevent true HPV infection by inducing and maintaining high levels of neutralizing antibodies. However, neutralizing antibodies against HPV does not control or eliminate existing HPV infection and/or transformed cells [47].

## Therapeutic vaccines

Therapeutic vaccines differ from preventive vaccines in that they rely on T-cell-mediated immune responses (specifically CD8 + T cells) that target the elimination of infected HPV and lesions rather than the production of neutralizing antibodies. Therapeutic HPV vaccines are facilitated by antigen-presenting cells (APCs), such as dendritic cells (DC). DC present HPV antigens via major histocompatibility class I (MHC-I) and class II (MHC-II) molecules, respectively activating HPV antigen-specific CD8 + and CD4 + T cells, targeting the killing of infected and/or transformed cells. E6 and E7 are not only near ideal targets for cervical cancer immunotherapy, but also the best targets for the development of therapeutic HPV vaccines. E6 and E7 are expressed in precancerous and invasive lesions and participate in cell cycle arrest, while they are not expressed in healthy cells. They are necessary to initiate and sustain HPV-related malignancies [47, 48]. Thus, therapeutic HPV vaccines against E6 and E7 are safe and can circumvent immune tolerance to autoantigens [47]. Most therapeutic vaccines currently in development include E6 and E7, where the DNA sequence encoding the E6 and E7 fusion proteins is inserted into the vector and mutations are introduced into the regions responsible for the interaction of E6 with p53 and E7 with pRB to reduce their carcinogenic ability. Various research teams are trying to develop a safe and effective therapeutic vaccine, and several HPV therapeutic vaccines designed to enhance the response of CD4 + and CD8 + T cells have been studied, including genetic vaccines (such as DNA/RNA/virus/bacteria), as well as protein-based, peptide-based or dendritic cell-based vaccines [49]. Numerous clinical trials have shown that some vaccines have great potential in treating precancerous lesions of uterine cervix. However, research on therapeutic vaccines against precancerous lesions has been slow, and no therapeutic vaccine has been approved for HPV infection and cervical precancerous lesions. Vaccine therapy has not yet been included in clinical guidelines, possibly due to the fact that vaccine development requires a greater human, material, financial and time investment, as well as many difficulties in terms of safety, immunogenicity, HPV polymorphism and mutagenicity [50–52]. In the future, further understanding of the molecular biology of tumors, further clinical trials, and the development of more effective therapeutic vaccines are needed.

## Prophylactic vaccines can be used as therapeutic vaccines

Continuous infection with high-risk HPV is the primary and only modifiable factor leading to the development of cervical lesions into HSIL and cervical cancer. Therefore, it has been proposed to vaccinate patients undergoing HSIL surgery with the preventive HPV vaccine. To date, no therapeutic HPV vaccine has been approved for HSIL. Attempting to vaccinate patients with advanced cervical lesions with the preventative HPV vaccine may be another simple and effective way to reduce recurrent HSIL and prevent cervical cancer. Del Pino et al. reported that 153 (57.7%) women in HSIL who underwent cervical conization received vaccination and 112 (42.3%) women refused vaccination. The incidence of persistent/recurrent HSIL was lower in vaccinated women compared to unvaccinated women [14]. Casajuana-Pérez A et al. reported that 563 HSIL patients underwent the cervical conization. The incidence of persistent/recurrent HSIL after surgery was 4.3% in the vaccinated group and 9.8% in the non-vaccinated group (HR: 0.43, 95% Confidence interval 0.22–0.84, *p* = 0.014). At follow-up 6 months after surgery, the persistence/recurrence rates were found to be very low in both groups, 1.1% in the vaccinated group and 1.5% in the non-vaccinated group (*p* > 0.05) [53]. A meta-analysis by Di Donato V showed that the recurrence rate of CIN1 + in Cervical Dysplasia who received the HPV vaccine during perioperative period was significantly lower than in those who did not receive the vaccine (OR 0.51; 95% CI 0.31–0.83; *p* = 0.006). The surgery reduced the overall risk of new or persistent CIN 2 + by 65 percent.The pooled estimated OR was 0.35 (95% CI 0.21–0.56; *p* < 0.0001) [54].

However, vaccination does not eliminate HPV that already exists in women [55–57], and there is still no conclusive evidence of the effectiveness of vaccination in patients after conization. In an analysis of a randomized controlled trial, it was found that no efficacy of the vaccine against cervical precancerous lesions was observed in patients who had cervical reactions after vaccination, and future randomized controlled trials are needed to further demonstrate this [58].

## Mechanism of preventive vaccines in preventing the recurrence of cervical lesions after cervical dissection

Vaccination can be used as a primary preventive measure against new HPV infections in previously unvaccinated patients, but no large-scale, multidisciplinary clinical trial has investigated the efficacy of the HPV vaccine for secondary prevention in patients with active HPV-related disease. The mechanism of action of the HPV vaccine in patients with pre-existing cervical lesions is not fully understood, and several hypotheses have been proposed so far to explain the exact protective mechanism of HPV vaccination in infected individuals. (1) The HPV vaccine induces the production of neutralizing antibodies against HPV capsid protein L1 virus particles. Neutralizing antibodies bind to newly acquired virus particles or virus particles produced by infected cells to inhibit infection of new cells by residual viruses, prevent HPV from entering host cells, and reduce the spread of existing infections. These vaccines do not effectively clear pre-existing infections because viral antigens are not expressed on the surface of infected cells and therefore cannot be targeted by antibodies after vaccination [59, 60]. (2) Cross protection against other HPV types [61, 62]. (3) Persistent HPV infection appears to be associated with changes in the local microenvironment and elevated levels of proinflammatory cytokines [63, 64]. From an immunological point of view, the presence of some inflammatory response in the local cervix after surgical treatment of cervical lesions is similar to that seen in patients without HPV infection. On the one hand, the vaccine has a protective effect against HPV infection in this localized microenvironment. On the other hand, the anti-inflammatory microenvironment is not conducive to sustained HPV infection, and surgical intervention to remove lesions from sustained HPV infection may be a good prerequisite for post-operative vaccine intervention [25, 26]. The HPV vaccine stimulates cell-mediated immunity, which may also play a role in preventing recurrent infections [65]. Therefore, HPV vaccination can protect women after cervical removal.

## Immunogenicity of prophylactic vaccines

Immunogenicity is the ability to generate an immune response and can be measured by antibody formation or cell-mediated immunity through cytotoxic T lymphocytes [66]. Long-term protection after HPV vaccination is important to prevent people from contracting HPV for life. Studies have shown that all HPV vaccines are highly immunogenic, with specific neutralizing antibodies against HPV antigens generated by HPV vaccination being 10–100 times higher than those produced by natural infection [67–69]. More than 98 percent of those vaccinated developed antibodies within a month of completing the vaccine, and they appear to provide protection for at least 10 years [70]. However, the level of antibodies produced depends on several factors:

### Sex and age of the individual and type of vaccine received

Several studies have shown that 9- and 15-year-old women have higher specific antibody titers than 16- and 26-year-old women who have been vaccinated [71–75]. A long-term assessment of the immunogenicity of the HPV16/HPV18 vaccine in serum from women aged 15–55 years showed high levels of seropositive antibodies against the HPV16 vaccine in all age groups 10 years after initial vaccination. In contrast, the positive rate of anti-HPV18 antibodies was higher in the 15–25 age group (99.2%) than in the 26–45 age group (93.7%) and the 46–55 age group (83.8%) [76]. However, in all study groups, anti-HPV16 and anti-HPV18 antibodies were higher than antibody titers produced by natural infection, and were predicted to persist above natural infection levels more than 30 years after vaccination [77]. As a result, young women between the ages of 15 and 26 are the primary target population for HPV vaccination [78].

### Adjuvant use and total vaccination doses

The addition of adjuvants improves the strength and persistence of the immune response. The bivalent vaccine contains the proprietary adjuvant ASO4, which consists of 500 mg of aluminum hydroxide and 50 mg of the Toll-like receptor 4 agonist, 3-O-desacyl-4 'phospholipid A, as an additional immune stimulant. 225 mg amorphous aluminum hydroxyphosphate sulfate (AAHS) as an adjuvant for tetravalent vaccines. Both vaccines have been reported to show effective safety and immunogenicity. However, bivalent vaccines yield higher immunogenicity against HPV infection than tetravalent vaccines [79–81].

### Active ingredients of vaccines

The concentration of each L1 VLPs and the antigen to adjuvant ratio are important differences between different prophylactic HPV vaccines, including Gardasil and Ceravix. Clinical studies have shown that the immunogenicity of three different doses of the preventative vaccine against HPV infection and cervical cancer is similar. However, levels of anti-PV16 and anti-PV18 antibodies were significantly reduced after vaccination with Gardasil compared to Cervarix [82]. Gardasil has twice the HPV16 L1 VLP concentration and the same HPV18 L1 VLP concentration as Cervarix. Gardasil-9 contains twice as much HPV18 L1 VLP, more than 50% as much HPV16 antigen, and twice as much Gardasil adjuvant. Therefore, in terms of vaccine immunogenicity, we should prioritize 9-valent vaccines, followed by 2-valent vaccines over 4-valent vaccines.

### Vaccination dose

Two doses of HPV vaccine are more protective than one, but different studies have not found statistically significant differences between two and three doses [81, 81, 83].

These results suggest that boosting vaccine doses and using appropriate adjuvants can improve the immune response and increase levels of neutralizing antibodies.

## Prophylactic HPV vaccines provide cross-over immune protection

The prophylactic HPV vaccine type elicits a specific immune response, and the structure of the L1 gene is similar to that of the non-vaccinated HPV type, resulting in long-term cross-reactive immunogenicity against the HPV type not included in the vaccine. Previous studies reported cross protection against HPV31 and HPV45 types after vaccination with a bivalent (HPV16/HPV18) vaccine. In addition, the cross-reactive immunogenicity of the quadrivalent HPV vaccine against HPV45 has been detected [84]. FUTURE I and II trials evaluated the cross-protection of the quadrivalent vaccine against 10 other HPV subtypes (32, 34, 36, 40, 45, 51, 52, 56, 58, 59). Vaccinating a portion of the infected population with the quadrivalent vaccine reduced the incidence of infection for up to six months, and CIN1 was associated with other non-vaccine-causing HPV types [85]. The PATRICIA and Costa Rica trials evaluated the cross protection of bivalent vaccines against persistent infection of non vaccine type HPV and CIN2 + , and found that the vaccines had higher efficacy against HPV-31, HPV-33 (two subtypes closely related to HPV-16), HPV-45 (closely related to HPV-18), and HPV-51 related infections and CIN2 + [86, 87]. Cervarix ® long-term follow-up data from the trial indicate that the cross-protection effect lasts at least 11 years [88, 89].These findings suggest that HPV vaccination may protect against new infections by providing cross-protection against non-vaccinated HPV types as well as other strains of HPV not previously exposed.

## Time for HPV vaccination: pre-surgery or post-surgery?

Post-operative HPV vaccination in women with HSIL can reduce the recurrence rate of lesions [19, 90]. However, the optimal timing for HPV vaccination has yet to be determined. Some studies have suggested that it may be more beneficial to get vaccinated before treatment. A recent follow-up by Sand FL in Denmark of more than 17000 women undergoing resection for HSIL found that women vaccinated before conization (between 0 and 3 months) had a lower absolute risk of developing HSIL compared to women who were not vaccinated, and women vaccinated after conization (0–12 months) had a similar risk of developing HSIL compared to women who were not vaccinated [91]. This is consistent with the results of Henere et al. [92]. There is growing evidence that pre-vaccination may improve patient outcomes [91]. Vaccination before conization ensures that the cervicovaginal area at the time of removal has sufficient anti-HPV neutralizing antibodies (removing most of the infected cells) to prevent re-infection of basal layer cells [93]. However, results from different studies were inconsistent. Saftlas et al. demonstrated that TNF-α in patients treated with LEEP was immediately reduced to levels similar to those in untreated controls [25]. Since surgical treatment induces changes in the microenvironment of the inflammatory tissue similar to those seen in patients without HPV infection, this may be a good prerequisite for post-operative vaccine intervention. A recent meta-analysis involving 11 studies and 21,310 patients showed that the HPV vaccine reduced the risk of relapse as an adjunct to CIN therapy. In seven of these studies [54], the first dose of vaccination was administered within the first month after surgery. Two studies reported that the first dose was administered between 3 months before and 12 months after surgery, while the other two studies did not specify the exact timing of the first dose [58, 91, 94, 95]. Due to the lack of standardization in the timing of HPV vaccination, it was not possible to compare the rates of CIN recurrence in women vaccinated before and after surgery. According to most studies, vaccination was given either before or shortly after LEEP/conization (up to 1 month after LEEP). Therefore, vaccination before or after surgery is recommended as soon as possible. Delaying vaccination may not prevent re-infection in women at risk of infection. In the future, we need to conduct further prospective studies and standardize the timing of vaccine administration to determine the optimal and most appropriate timing of vaccination to benefit more women and reduce the health care burden.

## Effect of HPV vaccination on women with persistent HPV infection following treatment

Continuous HPV infection after treatment, regardless of residual HSIL/CIN2-3, is the most significant identified risk factor for cervical cancer [96]. Another risk factor is the acquisition of a new HPV infection, as women who already have HSIL/CIN2-3 are at increased risk of developing new HPV-related lesions [97]. This is related to the persistence of lifestyle risk factors for HPV infection, which can persist for a lifetime, and these women are more sensitive to the persistence of HPV [98, 99].

Does the HPV vaccine benefit women who remain infected after treatment? Although the HPV vaccine stimulates local antibodies in the cervix that prevent the virus from entering the basal layer of the cervix, the effectiveness of prophylactic HPV vaccination in preventing widespread HPV infection has not been proven [28, 30, 100]. Some studies have suggested that the HPV vaccine may have some benefits in women with prevalent HPV infection [19]. Del Pino et al. [15] reported that women with persistent LSIL/HPV infection and even HSIL after the first coning also tended to have a lower HSIL persistence/recurrence rate by the end of follow-up after vaccination, but the difference was not statistically significant. A recent randomized controlled trial of 312 women with persistent SIL after conservative treatment of the cervix reported that HPV vaccination resulted in a more than 50% reduction in women with persistent LSIL or HSIL lesions [101].

## Dose and method of HPV vaccination

WHO initially recommended a three-dose regimen for HPV vaccination in 2009. A three-dose regimen is typical for infant vaccines based on inactivated proteins [93].

The second dose is given 1–2 months after the first dose, and the third dose is given 6 months after the first dose. The first two doses stimulate the production of immunological memory B cells in bone marrow, so they are called "initial dose". The second dose had an increased affinity for the antigen, resulting in a larger antibody response than the first dose. Due to this "affinity maturation" process, high affinity B lymphocytes are produced and differentiated into B memory cells that can quickly respond to antigens and produce NAbs [102]. Schiller and Lowy [103] showed that affinity maturation does not require multiple repeated doses. Because VLP is similar in shape to actual infection, sustained plasma cell production under reduced dose conditions ensures a strong and sustained immune response [102]. The Costa Rican vaccine trial team found that one, two and three doses of the bivalent HPV vaccine were equally protective against persistent HPV-16/HPV-18 infection over a four-year period and for up to a decade [104, 105]. However, the single dose vaccine was found to have a lower but stable antibody response [80]. In the CVT and PATRICIA trials, researchers demonstrated that the protective effect of a single dose of bivalent HPV vaccine persisted after seven years of follow-up. There were no cases of HPV infection during the entire follow-up period in the single dose group compared to the control group, which had an infection rate of 6.6 percent [105–107]. Another trial of the vaccine in 17,729 girls resulted in robust and sustained immune responses against HPV-16 and HPV-18 after a single dose. However, antibody conversion rates were significantly lower than in patients who received two and three doses of the vaccine [108]. The above studies suggest that a single dose of bivalent HPV vaccine may confer adequate protection [105–109].

Reducing vaccination doses due to financial and infrastructure constraints has significant public health implications, alleviating financial and logistical barriers to the introduction of HPV vaccination programmes. Single dose regimens will significantly reduce supply and storage issues and improve compliance. In recent years, multiple studies have evaluated the efficacy of shortening vaccination time in adolescents [110–114]. There is also tentative evidence that a two-dose regimen may also be appropriate for adult women [113, 114].

As a result, in 2014 the European Medical Association recommended only two doses of HPV vaccine for adults, [114, 115]. In 2016, the Advisory Committee on Immunization Practices announced that only two doses of the vaccine would be required for people younger than 15. The UK Joint Committee on Vaccination and Immunisation (JCVI) recently recommended routine use of the single-dose HPV vaccine in adolescent and MSM vaccination programmes. However, it is recommended to use a three-dose plan for vaccination of women aged 15–45 (at 0, 1–2, and 6 months) [83, 116]. Patients with weakened immune function should follow a three-dose regimen, regardless of gender and age at the time of vaccination [117]. Researchers are still exploring how and when to administer the vaccine to maximize its effectiveness. In essence, vaccines can be given multiple times by intramuscular (IM) or subcutaneous (SC) injections after a fixed time interval to stimulate the immune system and produce antigen-specific antibodies [118]. The skin is the first line of defense and is made up of large numbers of immune cells. For example, Langerhans cells in the epidermis and dendritic cells in the dermis can block the entry of pathogens and effectively absorb antigens to activate the immune system [119]. In a preclinical study, sublingual administration of the HPV16 L1 protein vaccine to a mouse model showed significant production of mucosal secretory IgA and serum IgG compared to other delivery methods, including intranasal, vaginal and percutaneous delivery [120]. Alternatively, it would be ideal to dehydrate the vaccine to make freeze-dried powder, transport it in powder form, reinsert it, then inject it in powder form or administer it intranasally [121].

## Side effects of HPV vaccination

According to the WHO Global Advisory Committee on Vaccine Safety (GACVS), HPV vaccines are classified as extremely safe [122]. The most common adverse reactions include swelling, pain, and redness at the injection site, but are usually short-lived and reversible. These reactions may be related to inflammation associated with VLP. Although it can immediately awaken the human immune system, its applications are limited [123, 124]. The systemic reactions of HPV vaccine include dizziness, headache, myalgia, fatigue, fever, vomiting, nausea, and diarrhea [123], with fatigue and headache being the most common (50–60%) [125]. Few other reactions were observed after vaccination [126]. While several case reports have described diagnosis of primary ovarian dysfunction following vaccination with the tetravalent HPV vaccine, there is no evidence to date to support a causal relationship [127]. The safety of vaccines in the progression of autoimmune diseases, venous thromboembolism, and neurological disorders has been studied in a variety of contexts, but no association has been found between HPV vaccines and these diseases [128]. In vaccine clinical trials, there was no difference in the rate of serious adverse events between the vaccine and control groups [126, 128]. National surveillance data following the introduction of the bivalent vaccine (Cervarix) and the quadrivalent vaccine (Gardasil) have shown lower rates of serious adverse events [128]. Most of the available surveillance data on the nonavalent vaccine (Gardasil 9) is from the United States and suggests that the nonavalent vaccine has a similar safety profile to the quadrivalent vaccine [129]. There have been no reported deaths from HPV vaccination [126, 129, 130].

## HPV vaccination in men

HPV is transmitted between men and women through sexual intercourse, and female patients may be re-infected by a man after the HPV has subsided. Sexual intercourse with condoms can reduce the risk of transmission, and the most appropriate method is to vaccinate both men and women against HPV to reduce the risk of infection [131]. The HPV vaccine was publicly funded in Canada in 2017 for men ages 9–26 and included in a school-based vaccination program. In 2019, HPV vaccination is recommended as routine for all U.S. men and women ages 9–26. However, since the introduction of the vaccine in men, adherence has been surprisingly low [132]. According to a 2017 systematic review, HPV vaccine adherence among men was only 47%, well below the Canadian government's target of > 85% [132]. Similarly, in the United States, about 44% of teenage boys (ages 13–17) are vaccinated [133], while among male college students, 53% say they have been vaccinated [134]. Other studies have shown that HPV vaccine completion rates among men (including a series of three HPV vaccines administered over a period of 6 months at the time) were only 14% [135]. Laserson AK et al. summarized the latest literature on HPV vaccination in college-age men and pointed out that the lack of understanding of the use of HPV vaccine and the lack of perceived risk and susceptibility to HPV infection were the main reasons for the low hpv vaccination rate in men. Sexual health education and advocacy campaigns through HPV sexual health education for men are key to addressing this issue. There is little literature on HPV vaccination in men to prevent the recurrence of cervical lesions in women, and large prospective studies are needed in the future to demonstrate this [136].

## The status and difficulties of HPV vaccination

Since the HPV vaccine was first approved in the United States in June 2006, a large amount of real-world data has proven that HPV vaccination is safe and effective in preventing and treating HPV infection and related diseases [129, 137, 138]. However, many low-income and middle-income countries have been unable to introduce HPV vaccines into their national immunization programmes due to financial and infrastructure constraints. Global coverage of the HPV vaccine as of 2020 remains low, with only 12% of women receiving two doses [139]. As of March 2022, 60% of WHO member countries have included the HPV vaccine in their national immunization schedules. HPV vaccination has been introduced in 114 of 145 upper-middle-income countries (78.6%), compared with only 20 of 80 low-and middle-income countries (37.5%) that have introduced national HPV vaccination [140, 141].

HPV vaccination rates have an important impact on cervical lesions and cervical cancer rates. Thus, addressing barriers to vaccination is key to achieving the full benefits of vaccines. Currently, there are still multiple barriers to HPV vaccination programs, including the following:

### High vaccine costs

The cost of vaccines is a significant barrier, especially in low- and middle-income countries. Recommendations to address this are to add HPV vaccination to the childhood immunization schedule and/or in combination with other vaccines, but this approach is not sustainable without external support [142].

### Cold-chain requirement

Another huge obstacle to HPV vaccination in low- and middle-income countries is the cold chain preservation of HPV vaccines, which requires high costs for preservation and transportation. Cold chain supply alone accounts for 80 percent of the cost of the vaccine. One solution to this problem is to dehydrate the vaccine ingredients and freeze them in powder form for storage and transportation at higher temperatures [143]. Another approach is that thermally stabilized capsomer preparations will also allow for a greater degree of temperature fluctuations, thus reducing transport costs [144]. Unfortunately, none of these agents are currently available. In addition, a single dose regimen can reduce costs and supply and storage issues. Global vaccination rates are likely to increase as vaccine stability improves and vaccination costs decrease.

### Vaccine hesitancy

In most low- and middle-income countries, the public is unaware of HPV-related diseases and national vaccination programmes [145]. Due to the diversity of cultural/religious beliefs, many baselessly associate HPV vaccine use with sexual promiscuity [142]. A cross-sectional survey of parents completed in 2017–2018 found that safety concerns were the most common reason parents gave for not vaccinating their adolescent children [146]. The solution is a combination of sex education and government-driven health policies that help increase public acceptance of vaccines and address parents' concerns through appropriate community education campaigns [147–149].

### Covid-19 pandemic

Globally, the COVID-19 pandemic in 2019 and the closure of schools and suspension of routine immunization schedules between 2019 and 2022 disrupted health systems around the world. Twenty-five million children missed the 2021 vaccination, a 30 percent increase from 2019. Many schemes are still recovering from the effects of the pandemic [142].

A shortage of HPV vaccines, lack of insurance coverage [150], the need to provide accurate information about the safety and effectiveness of HPV vaccines, and the need to make the vaccines available to health care providers have hampered HPV vaccination worldwide [151].

## Conclusion

There is increasing evidence that HPV vaccination reduces the risk of recurrent cervical lesions in women with surgically treated HSIL, and prospective studies are needed to assess the effect of the prophylactic vaccine on recurrent cervical lesions.

The best time for perioperative HPV vaccination is before or shortly after LEEP/ conization (up to 1 month after LEEP). Vaccination as soon as possible can better prevent reinfection in women at risk for HPV infection.The HPV vaccine is highly safe with few side effects and excellent immunogenicity. A single-dose administration regimen showed significant protection in susceptible populations, but its cross-over protection was limited. Existing HPV vaccines do not prevent all types of HPV, eliminate previous HPV infections, and treat related cervical diseases. As a result, significant numbers of people remain infected with HPV despite receiving the vaccine. In addition, multiple barriers to HPV vaccination currently keep global coverage low, particularly in low- and middle-income countries. There is an unmet need for therapeutic HPV vaccination, and HPV-associated malignancies will remain high for many years to come.

With our continuous in-depth understanding of the specific pathological mechanism of HPV and the molecular biological mechanism of tumors, the development of HPV therapeutic vaccines for various types of HPV immunogenicity, cheaper, less cold chain tolerance can be expected, and the goal of improving the coverage rate and success rate of the global HPV vaccine and eliminating cervical cancer will be achieved.

## Data Availability

All data grnerated or analyzed during this study are included in this published article.
